# On the Psychical Progress of Nations

**Published:** 1851-01-01

**Authors:** 


### Art. III.—ON THE PSYCHICAL PROGRESS OF NATIONS.

"The internal development of mind, so far as it is historical, belongs as much as the external events of politics, to the department of human history."
*Schlegel.*

The history of a nation is the history of a collective mind, for it is that which projects and governs its actions. As individuals compose a community, biography is the record of its common psychological character; so that a complete review of the psychical progress of nations is indeed a history of the world.

In tracing this progress from its outset, we must of course find ourselves at once in a dilemma, as the obscurity of ancient history renders it difficult to decide where the apocryphal ends and the authentic begins.

The pages of sacred writ, abounding as they do with symbol and allegory, require the deepest study and attention ere the divine can presume to expound and interpret their grand and awful truths. Man is introduced to us at the dawn of creation as a pure and innocent being, fresh from the hands of his Maker, an emanation, indeed, of the divine essence, endowed with qualities of which, as they partook of the angelic nature, and were fraught with holy intelligences and inspired knowledge, we can merely form conjecture.

Even for ages after the fall, we are told that there was immediate and special communication of the Creator with man, which, as it implies a subversion of those natural laws that, as it seems to us, now govern the universe, have been termed special or supernatural. The history of the antediluvian world, therefore, can no more form a part of our essay than the fables of Confucius or of Ovid.

Early profane history is so emblazoned with myth and exaggeration, that the selection and sifting of their truths is a work of still greater difficulty. The origin of almost every race is shrouded in obscurity—every ancient country had its mythical period, so that almost every historian of olden time opens his volume with a romance. The professed tale-writer returns the compliment in kind, by so blending fiction and truth, that shallow readers are completely bewildered in the maze—the more truth-like the fiction, the greater of course will be the dilemma. The plays of Shakespeare and the novels of Scott may in after ages excite as much controversy as the chronicles of the Cid, the poems of Ossian, or the epics of Homer. It is thus genius flings a gauze veil over the bright mirror of truth.

The psychical history of a nation, then, is a history of itself—its actions, its exploits, its manners, its literature, are the manifestations of its mind—the impulse of its psychical forces. For as it is with the being, so is it, from the *sympathy* of surrounding circumstances, with the nation of which he forms a part.

If the world had progressed in its primitive state of innocence, we may believe that a regular progression of the intellectual development of nations may have ensued; but rivalry, ambition, discord, war—the results of sin—have combined to thwart this Utopian march.

As nations and empires rise and fall, so of course the intellectual character of its members will flourish and decay. We must not, however, strictly measure the one by the other, nor must we always refer the political decline of an empire merely to a decadence of its intellectual energies. Young nations will again and again begin *de novo*—so that, however they may profit by the records and the wisdom of their predecessors, the universal mind is, as it were, broken up into sections, and we must often be content with tracing the psychical development of a nation as almost isolated from the influence of others.

Now if the globe were tenanted by one solitary being, it would be almost a folly to write about the development of his intellect. This tenant of the creation would, in the absence of psychical collision, be in every sense of the word a mere egotist—a slave to his animal instinct: his *mind* would ever centre in itself; for he would experience no blending of thought,—no excitement of the higher sympathies. Man would be a mere animal, with a superior or more extended capacity than other breathing things, and scarcely, perhaps, making a better use of his senses. However Camper might argue the accuracy of his facial angle, or Gall map out the surface of his cranium, still the facial mensuration would deceive, and the encephalic organization would, psychologically, be little more than a blank, in consequence of the want of influence to bring out its faculties.

If we would contemplate the immediate dawn of mind, and the relative influence of instinct and reason, we should perhaps study the isolated child or solitary savage; but these phenomena are most rare, and we must be content to watch the gradual unfolding of the mind, warped and modified as it ever will be by the incessant sway which surrounding circumstances exert over its progress, and to reconcile, as far as we can, psychology with general history.

We must not pause to compare the impulsive power, the centrifugal force, the unvarying agency of instinct with our self-controlling reason, —nor to inquire how far Rousseau and Monboddo were justified in their libellous comparisons,—nor to discuss the *nature* of mind. This would be vain, for the deepest philosophers, from Aristotle and Plato to our time, have differed, widely differed, in their psychological speculations. We must, however, be at once convinced that this mind is *a germ to be developed.* The savage, as Reid has hinted, may have the seeds of a logician or a saint, which only wait to be vivified, somewhat as the hatching of an animal ovum. Thus Peter the Great, from his bright and vivifying example and legislation, brought out all the intellect of the Muscovite boors, which otherwise would have yet been but a latent germ; and made his Russia one of the most powerful nations of the earth.

This national mind is often thus pent up, as it were, and not manifested—waiting for its exciting cause—an agency most varied and subtle, and immensely complicated. The spring of some of our own commotions was a mere expression of a man of high influence; and that of the second French revolution, the obstinacy of a citizen-king. So that when this spark of mind is blown on, it will burst forth like wildfire, in consequence of its psychical sympathies or imitative propensity. Witness the epidemics of the middle ages,—the Carbonari,—the Illuminati,—the Mormons, &c. &c. And all this may be pathologically explained; for the brains of all are excited, the disease becomes epidemic, and while it rages they call it an age of reason; soon, however, to be terminated by the downfal of debased tyranny. Even now millions of minds are so imprisoned, and only wait *an occasion* to burst their bounds. The late simultaneous overthrow of thrones and dynasties proves that the seeds of rebellion are universally scattered.

The progress of social life is one train of antagonisms. Instinct and reason are constantly warring against each other. There are infinite shades of difference displayed in this struggle—even in individuals of the same family,—and what is true of a tribe, is true, on a larger scale, of a nation.

We must not, however, reason *abstractedly* on this point, otherwise we might conjecture that two beings, placed in the same circumstances, would display the same traits of character—that the copper Indian might go hand-in-hand in the march of intellect with the Mexican and the Peruvian. The science of ethnology must be deeply studied ere we proceed to a disquisition on the psychical progress of mankind. The primal seat, the migration and the present locality, of every race should be contemplated,—a study in ethnological science which the maps of Pritchard and the erudite history of Latham* will tend to elucidate.

The various conditions of the brain, also, must be demonstrated, ere we can presume to a full analysis of human action and the manifestation of mind, or even to laud a fellow-being for his virtues or his vices. We know that cerebral conformation, even of individuals of the same race, assumes an infinite variety, and exerts a most potent and varied influence over the heart. Indeed we may, as moralists, venture to affirm, that it

* The Natural History of the Varieties of Man. By Robert Gordon Latham, M.D. F.R.S. London, John Van Voorst, Paternoster-row.

is almost as difficult for some to be evil as for others to be good. A seeming virtue may scarcely have a motive, and vice may be so irresistible as to call forth, not an anathema, but even our pity, to temper our judgment. Not that we would foster the maudlin mercy so much in fashion, that qualifies every dastardly attack as the irrepressible impulse of a maniac; but we would soften down the asperity with which we might be disposed to deprecate the acts of those whose natural temperament renders them morbidly prone to a course which others less excitable, and even their own cooler judgment, would deplore and condemn.

We do not in this argue the all-in-all of organization. Education may raise high a low capacity, and bad habits depress lofty intellect. The truth lies midway between the sceptics and proselytes of phrenology. The scientific study of ethnology has proved to us the value of comparative phrenology in our analysis of national character. Not that we presume to solve the problem once proposed—"Given, a physical man, to find the true intellectual scope of genius;" but we lay it down almost as an axiom, that intellectual quality is at least intimately associated with the relative proportions of cerebral to the rest of the nervous tissue. But this rule, even among the lower varieties of our race, must be taken with exceptions. We find the idiot, the *acephalous* monster, born among the most perfect of the European races; so do we find an occasional elevation of development even in the wilds of Æthiopia, with its corresponding psychical superiority. But these may be deemed the *lusus naturæ,* and, perhaps more than aught else, prove and illustrate phrenological truth. The divine indeed has based his *anti*-phrenological arguments on the faith of these exceptions, forgetting that there may be an exalted as well as a debased phenomenon of nature.

Then, although the capacity may be indicated by high frontal development, we must remember that the *tissue* of the brain may be *abnormal*—either the hemispherical ganglion that conceives or regulates the thought, or the tubular neurine that transmits it, may be diseased; and we believe that many psychical phenomena may be explained by a mere want of balance between these portions of the brain—in opposition somewhat to the hypotheses of our late friend Dr. Wigan, with whom we have had the gratification of exchanging thoughts and books.

Cowper's grey-matter, for a whimsical instance, might be firm and originate a healthy idea, but the soft medulla might soon feel fatigued by its transmission; while Walter Scott's well-balanced organization carried on its work with energy and harmony. Perchance some of these deviations may be referred to the parental age, state of mind and body, at the moment of impregnation, a subject which, although we do not follow up, engaged even the attention of that shrewd lady, the Margravine of Anspach. The more the capacity of the cranium, then, exceeds that of the face, we may look for a higher grade of intellect. The reverse of this, so characteristic of the darker races, is not more conspicuous than the inferiority of their psychical character—especially in the inhabitants of South Africa and the Polynesian islands, and the Arctic or hyperborean regions : and it is further illustrated by the facility of their extermination by the white races.

The aborigines of Australia, of Papua, of Congo, and other African districts, and even those of New Zealand, are characterised by the most degrading sensuality. Many of the southern Tasmanians have no idea of a deity, and their lives are one course of cruelty and crime. A mere dream is both an impetus and an excuse for the most diabolical act.

It is true that Barrow, Park, Barbot, and others, have eulogised the mild and generous disposition and chastity (!) of the black races, even of the Hottentot and Bosjesman, and have stated that they lend a willing ear and are easily converted by the missionaries. But *even if it be so*, their generosity is as much a blind impulse as their revenge—it is certainly not philanthropy. The Polynesian as well as Æthiop disregard with the most ungrateful indifference the valuable treasures which nature has scattered round them, and especially the bounteous fertility of their native soil. It is asserted, too, that some of the Bushmen, as well as the Esquimaux, are so stolid, that, like the Yahoos of Swift, they are scarcely worth making slaves of.

As we ascend in cranial proportions, we see corresponding psychical development—the Malay, and many of the Americans, and the insular Mongols are still inferior. Of the natives of Java, as a wondrous exception, Sir Stamford Raffles tells us they have no penal laws, because they have no crime !—resembling in this the Pimos of Western America; who, as Father Font informs us, neither steal nor quarrel, and therefore need no criminal or civil tribunals.

The American Indians, Dr. Von Martius asserts, are the lowest in psychology—but this may be explained by Robertson's affirmation, that the Spaniards petted their negro slaves, and thus reduced the status of the Indians; the Chaymas, and the Darien people, however, have to this day scarcely any idea of the science of numbers.

Even the Mexicans and Peruvians, together with psychical obtuseness, possess a corresponding apathy or want of sensibility, to which the term courage has been applied. Robertson eulogises the Mexican *drawings* with coloured feathers; but, like the Chinese pictures and carvings, they were merely mechanical and of fine colours, indicating—as do also their *casas grandes*, as well as the temples and palaces of the Incas in Peru, and the elaborately carved temples with pinnacles which Cordova discovered in 1817, among the Maya race, and which now exist in Yucatan—not a spark of *genius*.

When Cortez, with 500 men, came to Montezuma, although his city was immense and his court magnificent(?), and when he erected the Cross and the Virgin and destroyed the idols of Zempvalla, this legion of warriors made no resistance. Their language, however, is marked by an unity that proves their depressed character not altogether depending on psychical causes. Historians have also eulogised the lofty virtues of the Incas, and Molina especially of the Araucans of Chili; but this again is more apathy than noble endurance, a quality which is exhibited in the coldness of their passions. In such a clime we might suppose their love to have been a lava flood instead of that indifference, in which indeed they resemble their more savage neighbours, among whom women are degraded and despised, treated as slaves, and slighted to that degree that few of them are prolific, and if the mother dies during lactation her child is buried with her.

The savage and the negro especially are *conscious* of their inferiority, and contemplate the accomplishments of the white races with wonder and envy, and without the slightest hope of imitation. Mungo Park especially alludes to this feeling in his account of Pisonia. When Columbus, too, first landed on Hispaniola, the natives thought he was a god, and worshipped him. This sentiment seems also to have infected the natives of Oriental India; else the myriads of Hindus, especially those of the Sunderbunds, would not have quailed before a handful of Europeans, with which Clive and Hastings began and completed the conquest of Hindostan. And yet they were among the descendants of that people who, under the command of Porus, drove Alexander back from the Punjaub.

Now individual examples of psychical excellence have, though rarely, shone among the inferior races, as we see cretinism and idiocy among the most enlightened communities. We are now visited by the converted chief of a tribe of Ojibbeways, who speaks our language fluently, eloquently, and has conceived and laboured in the grand project of civilizing the Indians of the North-West. But these, as we have before hinted, are exceptions to the rule—otherwise Capitein, Omai, Feodor, and Thay Endanega, would not be cited as phenomena among the Æthiopian Malay, Mongol, and American races. They are not *types* of their races. Strange is it, then, that arguments should be adduced from this against a science on the ground of *exceptions*.

We must believe, then (whatever Humboldt and others have written to the contrary), that certain races of men are physically deficient in the highest attribute of our being, reason; and especially regarding the analysis of the relation of psychical impressions with externals. The minds of many of them are, therefore, cyphers—they are animals of sense and sensation, and little more,—reasoning, like a dog, merely from memory. They can scarcely conceive of mind distinct from matter, or trace more than the *immediate* step from an effect towards a cause; and, therefore, they of course stop far short of first truths.

Their thought is a vain dream; and as they have little power of controlling or directing it, their intellect may be deemed a mild insanity, guideless and wandering, without the faculty of attention, the basis of all mental exertion. In foresight, for example, how deficient is the Carib, who will sell his hammock in the morning, when refreshed by sleep, not dreaming that he shall need it again ere night comes on; and will neglect his sheltering hut when winter is gone.

Our chronological tables scarcely allude, in matters of science and literature, to others than the Caucasian variety of the old arrangement, and a few of the Mongols. Some of the Transatlantic tribes are endued with very acute sense and energy; but these qualities, any more than the stolidity of the Malay, and the grovelling nature of the Æthiop, come not within the category of intellect. These races, in different degrees as it were imperfect, constitute four of the five varieties, in the favourite classification of Blumenbach, and two of that of Cuvier, which was composed of three; the six varieties of the Systema Naturæ of Linnæus being a geographical error. In the varieties of man just quoted, the tripartite division combined with the ethnographic system of Pritchard, is nearly preserved *mutato nomine;* save that some of the psychically superior classes, as the Turks, the Magyars, Circassians, and Georgians, are blended with the Mongolidæ, the Japetidæ, European, and Asiatic, being pre-eminent in psychical excellence, and withal, with the exception of the Ugrians and the Turks, the conquerors and masters of the world. The researches of Dr. Latham are acute and laborious, and will have the effect of simplifying the science of ethnology, and of reconciling the psychical and philological history of mankind.

Regarding this psychical depression, we may refer, on the ground of analogy and of historical record, to one influential cause, especially observed among the Jews, and some other races, who, from principle, custom, law, or isolation, are *exclusive* in their generation. The Negro, if distaste did not so often counteract it, might somewhat improve his breed by crossing—indeed, we *do* see both beauty and intellect advanced by this intermingling of races. The infusion of European blood into the savage races at once raises the psychical character. The Mestigo, or cross between the European and the American Indian, is of higher intellect than the Mulatto, the offspring of a white and a Negro, and, indeed, little inferior to the Creole, who is of European blood, born in a Transatlantic clime. My esteemed, and I fear lamented friend, John Franklin, assured me, that the psychical qualities of the half-breeds of North America were far superior to that of the races devoid of European blood. The Persian nobles thus improved both the physical and psychical qualities of their races, by alliances with their Georgian captives. If we contrast the intellectual beauty of the Persian nobility with that of the Peninsular, who are almost exclusive in their unions, we shall at once be inclined to refer it to the excellence of Circassian development. Their breeding *in-and-in*, we may believe, has often depressed both the constitution and intellect of royal and noble families, as well as of the Hebrew race. The system of crossing, indeed, is a *lex scripta* among the breeders of flocks and herds—to which, perhaps, the vigour of bastardy may offer some analogy;—the dwindling also of the potato tuber is certain if it be limited to one locality or soil.

If we measure psychology on geographical principle, we shall discover that the seat of science has been limited to the temperate zone, and has ranged between the 35th and 60th degrees of north latitude. And here, also, we have the clearest proof of the importance of organization in psychical advance, displayed in the comparative intellectual powers of different races within these degrees; those of central Asia, for instance, compared with those of Europe. Even among the Chinese, the highest of the Asiatic Mongolidæ, slavery is the fashion, from the chief mandarin, downwards.

There is also something of an interesting assimilation between the corresponding latitudes of the old and new world. Of this the Mugrisians, the Samoiedes, the Ugrians, the Fins, the Kamschatkadales, and the Esquimaux, are examples. The Tartar corresponds with the Mexican, the Moor with the Floridan or the Natches, some of the least unintellectual among the Transatlantic aborigines: the Malay with the Yucatan; the Papuan with the Carib; the Albinoes of Darien with the Negroes of Africa, among which, indeed, are observed whole families of white Negroes; these, however, are mules or abortions, rather than a race.

If we compare the varieties of man, regarding their power of resistance, even among conquered nations, we find them greater in the Caucasians, and also their faculty of imitation or acquirement of knowledge from their victors. The Britons and Celts soon profited so much by the contemplation of their invaders, that they eventually turned and dislodged them. The Polynesians have never learned this lesson from *their* conquerors. These aborigines, evincing little more than a tutored instinct, continue to drudge and grovel in their slavery, and will, in the end, be exterminated. The Tasmanians, located in one of the most luxuriant districts of the globe, had not begun to till the soil long after the Europeans had settled among them and were reaping the rich harvests of their cultivation.

Mere isolation cannot sufficiently account for all these deficiencies, this tardy progress or abeyance of intellect. No doubt the collision—

the antagonism of intellect of an inferior with a superior race, will often confer on it great improvement. The step of the ancient Romans to eloquence and refinement was the example of Greece and Sicily. With nations of the same degree of capacity, this collision will ever prove a mutual benefit; for the intellectual races of the world are like the learned societies of a metropolis—one communicates with the other, and both discuss, and thus mind and truth are developed, the germs of knowledge are brought into light. As one field lies fallow another is tilled; so the crops of science are perpetually springing, and new minds are added to the intellectual world.

The immense facility of present intercommunication will speedily effect wonders, and equalize and assimilate all the races displaying an equal degree of cerebral development. The Georgian frontal beauty may not long want the corresponding grade of intellect, yet it has hitherto been so. But Minerva may yet rebuild her temple in Western Asia. It may be that some approximation of physiognomical expression may also be effected by this interchange and culture of mind; for as there must be organic development to ensure intellect, intellectual study will modify the cranial proportions, so that in the end an untutored European may display less intellect than a cultivated Asiatic Mongol.

With cerebral development we might here combine *temperament*, age, and sex—all deeply influential in the creation of *character*. The first only comes within our limits. The constitution of the blood, as we see it indicated, especially by hue and complexion, bears intimately on the subject. The terms melancholic, nervous, lymphatic, and sanguine, are but definitions of condition dependent chiefly on the blood and its products. Yet, although they are of national as well as individual importance, we must leave them among the principles of *general* pathology. Not the least effect of this intercommunication will be its influence on language, to which psychical progress must be so much indebted, and by which it may indeed be in some degree measured. If we regard the Polynesians,—almost all the Oceanic Mongolidæ of Latham, except those of the Malay peninsula, and especially the Papuans,—we find the language one of almost inarticulate gabbling, resembling, even in the opinion of the peninsular Malays, the chattering of birds. In them there is no psychical progress; their faculty is like instinct, as it was in the beginning.

Even the Chinese and the Tibetian, among the Altaic Mongolidæ of Latham, though ingenious and clever, are certainly not intellectual; the language is monosyllabic, aptotic, and puerile; the alphabet, which is hieroglyphic or rhæmatographic, consisting of 80,000 words. We cannot believe that there can be a lofty psychical progress when a dictionary can scarcely be learned or comprehended in a life.

The other monosyllabic languages are those of Oriental Tartary, the Malayan, and Indo-American districts, and we see at once their association with a low psychical development.

There are, however, many glossological problems to be solved. The Yakuts, or Northern Asiatic Mongols, who resemble the Esquimaux in character, possess a language intelligible in Constantinople. The polysyllabic tongues indicate a race of higher intellect. The Indo-Persic, the Græco-Latin, the Teutonic, with the Arabic and Hebrew, are clearly and excellently adapted for the communication of ideas, and with them we witness the perfection of intellect. The language of the Circassians, however, once deemed the model of a race, is not perfect—"position," according to Dr. Latham, "doing the work of an inflection," —as *ab-ăcē*—*father-horse*—the *priority* of *ab* indicating possession, or the genitive case. The roots of language, however, may often be almost forgotten when we are perusing the perfect *glot.* The various languages comprehend five or six thousand families, split into innumerable dialects or idioms seemingly dissimilar. Even those of Britain are five or six; the Teutonic or German, the Scandinavian or Norse, and the Gaelic and Cwmric branches of the Celtic, contributing to perfect our modern English tongue.

We cannot attempt to analyse the system of glossology. Among the families of Asia and Europe, however, very striking resemblances are discovered between tongues at first apparently dissimilar, *e. g.* those of India, Persia, Greece, Rome, Germany, and perhaps the Scandinavian or Norse, the Slavonic and the Celtic being still dissimilar; these comprehend the intellectual races. The Tschudic, the Oceanic, and others, are the medium merely of conversation and commerce. Regarding the intermingling of races also, we have in glossology a proof of intellectual superiority: the most perfect or psychical language soon becomes the common medium, as we see in the modern languages of this day. We may yet refer all inflectional languages more or less to classical roots. To analyse these would require a volume; and perhaps, in attempting such a labour one might often come to this quaint conclusion of Goldsmith—by changing "Psam" into "A," and "mis" into "toes," we prove, by a very natural and easy conversion, King Psammis and Atoes to be one and the same man.

The analysis of Dr. Latham, however, is so acute and laborious, that we may safely refer the curious philologist to his erudite volume. The classification is more simple and more *natural* than that of previous systems, (the tripartite division of the human race being that which we ourselves have long mentally adopted,) and it will assuredly form the text-book of future disquisitions in ethnology.

We believe, then, that we may consider organization as the *natural*

essence of psychical development. The earliest history of mankind, sacred and profane, refers with scarcely an exception to that race hitherto termed Caucasian, including the Hindus of high caste, the Persian, and the Egyptian. With these we witness the dayspring of the arts, the dawn of early literature.

Sculpture was confined to the schools of Greece, Italy, France, and England, and painting to the latter three. The national mind of their great cities has long gloried in the pre-eminence of their art. The Florentine, like the ancient Athenian, looks on his glorious city with almost a filial veneration. The magnificence of the Oriental temples, Salsette, Elephanta, even Thebes and Carnac, does not indicate so high a degree of development; its beauty is merely that of gorgeous and exquisite *detail;* it possesses no architectural proportion or symmetry that approaches the styles of the gothic or classic lands. We allude to India, China, and Persia, and not, of course, to Palmyra and Balbec.

The pillars and huge structures of Egypt, the pyramids, Memnon, and the Sphinx, like the Cyclopean works of Greece, the colossal statues that La Perouse and Cook discovered in Easter Island, and our own Stonehenge, certainly excite our wonder, but it is the admiration of *magnitude:* they are the effects of slave-labour, the co-operation of a servile clan. The newly discovered Transatlantic temples, especially those of Yucatan, which, as they closely resemble the Oriental, indicate an ethnological mystery yet to be unfolded, are *curious*, but not beautiful. Regarding India especially, perhaps the division of the nation into servile castes may have somewhat to do with this. The Brahmin was ordained to pray and teach, the Chehetree to fight, the Bice to till and traffic, and the Sooder to serve and labour. If he violate these rules of his caste he is excommunicated and outlawed, and is called a Pariah or a Chandela. Thus the Hindu mind has been fettered, whatever his genius, to *one line* of exertion, and he must of necessity lag behind the freeborn European, whose genius is allowed full scope to soar as his enthusiasm may direct or impel him.

It has been affirmed that we are now below the ancients in the march of science. Guizot has asserted that "modern literature is far inferior to the ancient." Is it so? No; our modern works are little short of a miracle, and if the magic of engineering slept with Archimedes, (and we must accept no more than a tithe of his greatly-exaggerated power,) Smeaton and Telford, and Brunel and Stephenson, have, with a glorious purpose, roused it from its slumbers. The works of their lofty genius are evidence of the *progressive* nature of intellect.

If, in the composition of colour, Correggio and Titian, and Claude and Murillo, may have been pre-eminent, the modern English school far eclipses them in conception, freedom, and delicacy of touch.

In the senate, the elegance of Cicero and the energy of Demosthenes have been at least equalled by many a modern statesman; and the Virgilian numbers must yield to the magnificent genius, the poetic beauty, and the exquisite verbiage of Byron.

The perfection of the wondrous creations, the universality of genius, of the twin stars, Shakspeare and Scott, (for it is no profanation to associate the bard of the Tweed with him of the Avon,) have gone forth, and are confessed, throughout the civilized world.

But to favour this pre-eminence, were combined geographical position, climate, temperature, soil; their blood was neither chilled into apathetic and mindless indifference by the icy breath of the Arctic circle, nor their temperaments disordered, their dispositions degraded, nor their energy dropped, by the hot winds of the tropics. Their lives were not, like those of the hyperborean races, one unvaried course of defence against elemental foes, nor of voluptuous indulgence, like those of the equinoctial or tropic people. The sun smiled on their climate like a mild and fostering nurse, rendering it beautifully varied and congenial.

They were, therefore, surrounded by circumstances calculated to foster social and intellectual development; there was a combination of favouring causes.

Tasso would not have recorded his voluptuous love-breathings amid the snows of Zemla, nor would the pencils of Raphael and Correggio have created their divinities had they set up their easels amid the sands of Sahara.

In the marshes we see the lymphatic temperament prevail, combined with indolence and mental sloth. Abdera was so characterized that stupidity was termed "Abderitica mens!" When we are compelled to resist an excess of evil—the stern realities of life—instinct, as in the Esquimaux, is called into play, rather than reason. In cold, heat, hunger, there is little scope for the poetry of existence. Eulogise as we may the *genius* of Burns, Bloomfield, Hogg—would they not have written better in the smiles of prosperity, in trimming that native fire which reverses could not altogether quench? For after all there was much common stuff emanating from their pens, and they eminently failed in farm-keeping and shoe-making. All that we can say is, "they were very fair *notwithstanding*."

As a rule, therefore, the intellectual being is more perfect if he breathes an atmosphere congenial to the expansion of mind: and a hue is often imparted to the *species* of intellectual blossoming and fruit by the especial qualities of climate. In the sunny clime of the Mediterranean, poetry and painting found a home. Cradled amid the shadows of the glorious Appenines, smiled on by the sun, o'ercanopied by the "deeply, darkly, beautifully blue" arch of heaven, the sister arts have been instilled into hearts and minds almost from infancy. Still would we not imply that mind is cold or barren in proportion as it approaches the Arctic circle. The Scandinavian Sagas, the Skalds of Iceland, Upsala, Stockholm, Gustavus, Linnæus, Berzelius, Jenny Lind, will vindicate the claims of the Baltic to all posterity.

Yet organization bears its sway, although the constitution of the mind and the constitution of the climate contrast and oppose each other. Circumstances may alter, they will not metamorphose the intellectual being. His superiority will loom out at the equator, "or Zemla, or the Lord knows where," while the Æthiop will never soar, although the Royal Society, and the French Academy to boot, were to take him in hand.

"Cœlum non animum mutant qui trans mare currunt."

Even the old Roman spirits do not sleep. The Trasteverini still boast of their Roman lineage, and even their descent from Troy; and their classic physiognomy does not belie them.

To attempt to trace the course of the human mind, from its dawn to the present hour, must be as futile as to adduce an abstract cause for every intellectual manifestation.

Regarding the very origin of a nation, it is clear, if we adduce Romulus, Brahma, or Odin, as founders, that history at once dwindles to a fable. The maps, too, are marked with the sites of illustrious countries and cities—Babylon and Ilium, for instance; yet the locality and limits of the first, as well as those of Assyria, Syria, Phœnicia, Scythia, and the very existence of the second, are still doubted. The date as well as the foundation of ancient kingdoms, and their relative priority of birth, must often be mere conjecture. We must, therefore, ever commence our task at a far later date than their affirmed birth. The early records of Egypt, Assyria, Chaldæa, India, China, Greece, Rome, are all involved in obscurity. The researches of Rawlinson and Layard may unfold much of this mystery.

Then the ancient and modern histories regarding religion and language must essentially differ. The Egyptians, once the subjects of the Pharaohs and Ptolemies, are now a jumble; the Arab hordes, once Sabeans, are now Mahomedans; and the Copts are Christians.

Throughout the earlier ages the heathen philosophy of the Hindu sages and the Egyptian priests was blended with the glowing psychology of the sons of Iran after the victories of Cyrus and Cambyses, imparting that beautiful though completely imaginative tone to the psychical character of the then known world.

We must also constantly separate the early and the late theology of nations. Osiris, Bel, Dagon, Thaumuz, do not now reign as the mythological gods of the Egyptians, the Assyrians, and the Syrians. The grand metempsychosis from imagination to rationalism took place on the field of Marathon, when the Hellenes overcame the fire-worshippers, and a colder philosophy, still blended with the myths of the Zend and the Sanskrit which tinge and taint our classic pages, was adopted. This may be termed the second grand spring of the human mind; for although Roman arms subdued the bodies of Greece, the psychical stamp of the Hellenic philosophy and poetry remains a model to our own day.

In the Oriental records we constantly discover the greatest ignorance and presumption. The Japanese affirm the reign of one of their emperors to have been more than two millions of years; and the Hindus boast the high antiquity of their Sanskrit, also to hundreds of thousands of years. The Chinese are equally bombastic, although the real history of their religion cannot date further than 200 years before the Christian era; while the Indian Sagas would locate the physical and psychical birth of the world in the immediate vicinity of that mountain range which, either on this account or that it is the geographical summit of the globe, they have denominated Himalaya, or Heaven.

Now, wherever intellect had its birth in the east, it is clear that its tide has set progressively westward. India, Persia, Chaldæa, Assyria, Judæa, what now is the manifestation of their psychical degree? slavery, bigotry, or ignorance; and the learning that was once their pride and glory has fled to adorn and enrich the climes of Western Europe.

Schlegel, with the peculiar intuition of his country, has compared, very graphically, the mental elements of the four chief nations of the primitive world.

The national mind of the Hebrews, he believes, was eminently susceptible of divine truth; that of Egypt, deeply constructive and skilled in the more abstruse mysteries of science,—as we learn, indeed, from the description of the golden zodiac in the temple of Orymandes, and other astronomical works; that of the Hindu is coloured (we may write stained) by the most prurient imaginativeness; while that of China is to this day, with all its idol-worship, the same simple theism as in the time of Confucius—the slavery of sovereign reason.

This might once be true, but there has been much amalgamation of these various attributes. There is much resemblance in poetic imaginativeness between the Indian, the Persian, and the Hebrew, while the Egyptian is completely metamorphosed.

The Altaic Mongolidæ are still unchanged. The Chinese alone—the "Mongol softened down"—although an isolated race, exhibit superior psychical character. Literature scarcely forms a department of their intellect; but their genius is extensively though not deeply blended with the arts and luxuries of the world, of which silk, tea, and porcelain are the daily illustrations. Their exclusiveness, isolation, narrow-minded jealousy, and the religion of Fo, which they profess in common with the offsets of the empire, mark them as dissociated from the rest of the civilized world. Yet the analogies of their sacrifices, their laws, both with other ancient nations and with the records of holy writ, point to some yet unfolded secret.

Their offerings resemble those of the first brothers in Paradise. In the countries bordering on Assam, one man is enjoined to marry his brother's widow; and a lover, as in the land of Israel, wins or earns his wife by years of agricultural servitude; while the patriarchal system is the prominent feature of family life. In some districts of the Thibetian country, polyandry is said to have prevailed; but the history of China and India, and Persia and Greece, exhibit so many close affinities, that ethnology may have an interesting study even in tracing their analogies and parallels.

The early records of the Greeks claim almost as high antiquity as the Hebrews. Indeed, they refer to their king, Pelasgus, who gave his name to the primitive inhabitants of Greece, as "Earth's firstborn," and who eventually overran Italy, Spain, and other Mediterranean lands. They had also a deluge and an earthquake, which separated Ossa from Olympus. Sicyon is called the first Greek city; its date more than 2000 years before Christ. Long after this, the Hellenes, the Danii, the Dorians, the Cadmians, the Argives, possessed the land. The age of the heroes Perseus, Hercules, Theseus, Jason, Achilles, their myths and polytheism, compose a fable, and we of course pass by their metallic "dome of heaven," and the author, whoever he be, who wrote the first and finest epics of any age—the Iliad and the Odyssey, and take up, as the first authentic instance of their wisdom, the confederacy of the Amphictyonic Council, and the sources of their high pride of prowess—the Olympic, Nemæan, and Isthmian games. Then follow the legislative wisdom of Lycurgus, in Lacedæmon; of Solon, in Attica; and the establishment of the first tripartite constitution of king, lords, and commons—then termed the Archon, the Areopagus, and the Four Hundred; and the school of Phidias, from which first beamed forth the perfections of ideal beauty. Let us but glance at all this, and we must confess the eminence of early Greece in aught that could ennoble and adorn a heathen race.

From 300 to 500 years before Christ, a resplendent constellation still cast a halo over Greece: Socrates, Plato, Aristotle, in philosophy; Demosthenes, in the Senate; Sophocles and Æschylus, in the drama; Phidias and Zeuxis, in the arts; Miltiades, Themistocles, Leonidas, in the battle-field; Hippocrates, in the science of medicine. The noble Pantheon was built by Pericles, and adorned by the magic chisel of

Phidias. These, and many other men and deeds of worth, must mark the palmy days of Greece as the most glorious æra of the world.

For a long period ancient history records a series of intestine and foreign wars. For two centuries, at least, there was a dearth of high psychical character: but an æra was approaching which was to throw a holy light over the path of man. Anaxagoras, the first Unitarian, had sapped the foundation of polytheism, and proved the being of one deity —the source of nature; Scipio, by the third Punic war, had destroyed Carthage, the mistress of half the then known Europe, and with her, the last of the great blood-sacrificers; and Plato, by his disquisition on the immortality of the soul, paved the way, as it were, for the Christian æra.

The triumph of peace in moulding the national psychology of Rome rendered the Augustine æra so illustrious as to become a proverb. Virgil, Ovid, Horace, Cicero, Livy, Tibullus, rendered their age the spring of that sparkling brilliancy that even now delights the classic mind; but the character of that early æra, like that of pagan Greece, was not virtue. Stoicism, it might be; but that was but another name for heathen pride. As yet there was no divine light to consecrate the mind and heart. Men were the *rational* pupils of Nature, and adored her with a blind worship—self-murder was a virtue! Even the honied numbers of Virgil and Horace were debased by licentiousness; and it is a melancholy evil that those pages, which abound with all the beauty, melody, and sublimity of poetry, should teem with the most debasing immorality.

After an age in which sloth or vice had infected the rulers of pagan Rome, and contentions inflamed the minds both of patrician and plebeian, the sun of Christianity arose, and as it purified and softened the hearts of men seems to have changed the psychical character also of the age. History and biography were the subjects of the pen—the gospels and the records of the apostles' acts engrossed the mind. The Plinys, Josephus, Tacitus, Florus, Plutarch, compiled their histories, until at length Constantine removed the seat of empire and learning to Byzantium, and spread Christianity to the shores of the Bosphorus. This transplantation did not produce much advance of intellectuality, although the Greek denizens who were called the Byzantine historians, have left voluminous records. And when the second Mahomet again conquered Constantinople from the Greeks, the downfall of all intellectuality ensued. The only evidences indeed of psychical energy or eminence being displayed in their architecture, especially the Mosques, as that of the Moors during their sojourn in Spain was by the construction of an Alhambra.

The Greek emigrants deluged Rome and Constantinople with Hellenic literature, and from thence the Western nations of Europe, Italy,

France, Germany, Britain, Florence, which subsequently, under the Medici and Bessarion, became almost Greek academies.

Aristotle and Plato were the great oracles of the schools of psychology and natural history, and the terms of art and science were defined by Greek names. It was the Latin language, however, that characterized all the northern shores of the Mediterranean, the Peninsula, and indeed all the European nations not originally Sclavonic, as Russia and Poland, and Illyria on the Adriatic shore, and the Roman alphabet, blended with the Greek, became the prototype of all the present alphabets of the intellectual world. Even when, in the 17th century, the Roman influence had waned, and Gaul, Britain, Spain, and Italy were the abode of Franks, Saxons, Goths, and Lombards, the classic language was still that of the church and theology.

We therefore regard Rome as the great centre or focus of intellect into which the Orientals, the Greeks, and the Germans poured their treasury of learning, to be concentrated at last in the west. At the time Gregory filled the papal chair his mind was engrossed by his missions. The chief occupation of his monks was the writing and illumining of missals, until Augustine infused the light of Christianity into the British people, a branch of that race which Dr. Latham calls Indo-Germanic Japetidæ, crossed with the Kelts or occidental Japetidæ, from their first seat in the central regions of Europe, the farthest point to which we can trace them.

The desire for learning which Augustine imparted to the Britons was more and more fostered by the Anglo-Saxons, (although the myths and mysteries of the Teutonics tinged for a time the mind of the British isles—as we even now discover in the derivations of our week-day names) until Alfred in his wisdom established the first English constitution, about the period when Charlemagne was endeavouring to enlighten France. It is curious that light poetry from the lips of his mother should have brought out the first truly great mind that shone upon the British isles.

The translation of a portion of the sacred Scriptures and of Bede's history, the endowment of public schools, and the mission of the first overland expedition to India, prove the enlightened scope of Alfred's mind. But Alfred had been twice to Rome, and had received the royal unction from Leo the Third.

Still his high example conferred no lasting mark on the psychical character of the Anglo-Saxons, whose intellectual attainments were slighted, and termed barbarian ignorance, even by the unintellectual Normans.

Before the Conquest, Britain was composed of three races, Anglo-Saxon, Gallic, and the Cwmri; a strange compound of Angles, Saxons,

Cambrian, and Pictish Kelts, Irish and Scotch, and Manx Gaels, and different districts of our isles were characterized by the Saxon, the Mercian, the Hibernian, and Caledonian idioms. In our own day, the Irish or Erse idiom is confined to their western counties and the lower classes of Erin—the Caledonian to the western isles and the Highlands, and Manx to the mining and northern districts of Man.

After the Conquest, the national mind was long agitated, especially by contentions with the Saxons, until the reign of Henry the First, which was perhaps the most quiet and peaceful one of British annals. It was not, however, until the reign of the Second Henry, the most accomplished and amiable prince of his time, that the psychical character of the British nation was first elevated to an intellectual degree. Even then the communication of ideas was little more than oral. Records were few, consisting of Anglo-Saxon runes, a few monkish missals, and the manuscripts of the clerici or clerks : although even in pagan Rome there was a sort of printing, and, as Roger Bacon informs us, a sort of block printing, even then in China. But there were no master minds to work out a discovery like those of Guttenburg and Caxton to perfect that art, which produced the greatest psychical renovation of the age, and which Luther felt eventually to be the greatest help in the march of the Reformation, especially by the spread of the Greek language, which he deemed essential to the study of the holy books.

After the feudal system and the great charter had civilized and quieted the people, the expanded mind of the third Edward raised still higher the prowess and the fame of England. The battles of Cresci and Poictiers were fought and won, and by the conquest of Wales and Scotland, England became Great Britain.

This was the period in which the Saxon and old English dialects became pure English. Chaucer's poetry, "that well of English undefiled," and Mandeville's Travels, being the first well-written books of the fourteenth century.

The wars of the Roses, an age of fraternal bloodshed, again blighted the national mind, and filled the land with weeping and groaning. Henry of Lancaster sat on a throne of thorns, and his son stained and desecrated his reign with his domestic murders. The virtues of Edward the Sixth again brought blessings on the land. In few brief years of his reign, three large hospitals arose, which still cast a glory round his name. England was a school of pure charity—too soon to be converted again into a bloody arena. Let us sum up the murders of the Catholic and the Protestant sisters. Jane Grey and Mary Stuart, Seymour, Somerset, Dudley, Northumberland, Norfolk, and Essex, for state policy; Cranmer, Ridley, and Latimer, for *heresy*. Let us blush for the dark side of human nature.

The age of Elizabeth, however, was studded with stars of learning, the greatest of which was Bacon, whose inductive philosophy, in opposition to that of Leibnitz on the continent, enlightened, while it strengthened and purified the national mind. It was then the fashion for ladies to be learned; the queen herself was deeply read, and not a little proud in boasting of her attainments. Sidney, Spenser, Raleigh—above all, Shakspear, complete the psychical glory of the Elizabethan reign. This was the end of what was termed the middle English language, and from this period there was a change, not only of language itself, but of national thought and sentiments.

During the Stuart dynasty, if we except the commonwealth and the belligerent reign of the Nassau, learning and wit were not idle. Boyle and Newton, Sydenham and Harvey; Dryden, Butler, Otway, Pope, Addison; and Buckingham and Rochester, attest the psychical eminence of Britain in philosophy, medicine, poetry, and wit.

To revert. The inundation from Scythia seems to have populated the greater part of Europe; to them the Cimbri or Danes, the Teutones or Germans, and the Scandinavian, or Norse, owe their origin. Yet among them we perceive superstitions that closely resemble those of the Orientals, and even the transatlantic Indians. The Keltic Druids, like the Hindus, the Peruvians, and the Carthaginians, burned victims on the altar, to propitiate their gods; and like the Pythagoreans, they believed in metempsychosis.

The early creed of the Norse races was theism. It was a religion of sensuality, like that of Mahomedans and of the classic mythology. Both in Odin's and Mahomet's paradise, voluptuous girls ministered to those renowned for earthly virtue. The valkas of Odin, however, were more of the Hebe; Mahomet's houris, more of the Venus. Drinking, therefore, was the order of the day with Odin—sensuality with Mahomet. Yet we are told the Goths taught the Southerns chastity. We doubt it. And it was seven hundred years after what they term the coming of Odin, ere they were Christianized.

Their psychical scope was limited, the Runic stones and sticks being almost the only aids to memory, and confined to the Scandinavian scribes, who little dreamed of making a volume to record a train of thoughts and reasonings. Their intellect was debased; the poetry of their scalds, or poets, absurd.

The discovery and settlement of Iceland and Greenland, however, are not unimportant points in geography, as it was indeed the first discovery of America, if we may credit the beautiful "Codex Flatoiensis," written on vellum by Eirek the Red, one hundred years before the first voyage of Columbus.

The pages of Icelandic literature certainly indicate some genius, the Eddaic being superior to the Skald and Saga writing; its style resembling in a low degree the magnificent Arabian romance of Antar.

Like the Bedouins, the European Slavi were originally wanderers, and were soon overwhelmed by Ostrogoths, Ugrians, and Fins. The offspring of the Slavonians are Russians of the Greek church; and Servians, Poles, and Illyrians, of the Romanist. Their psychical character was ever low, rude warfare being their chief aim; although roots of their language are interwoven with the classic. The spring of the intellectual advance of the Russian, was the establishment of the Greek church by Vladimir, and the baptism of the Princess Olga. The people had become half Christianized, when Yaroslof, the successor of this Russian Alfred, like Egbert of England united many provinces. Still, commotions and intestine war depressed the intellectual scale, until Peter I., by his own quaint though firm example, raised the psychical and constructive spirit of his nation, which Catherine, with all her grossness, still improved, herself being a dramatic writer. Yet they were a nation of serfs, until Alexander civilized the Russian boor by the foundation of schools and universities, and orders of merit. How did his country prove its gratitude? The czar, as we are informed by a noble lady, walked at his coronation, "preceded by the assassins of his grandfather, followed by those of his father, and surrounded by his own."

The first natives of the peninsula were Iberians; the Basque provinces, however, excite our greater interest psychologically, as their language bears a more ancient date than any other in Europe, being spoken not only throughout Spain and Portugal, but carried to the eastern shores of the Mediterranean. The peninsula has, however, been the constant victim of invasion. She was the prey of the Romans and of the Moors, when the Arabic was the common language, until Ferdinand and Isabella, the sovereigns of Aragon and of Castile, terminated the wars of the cross and the crescent, and made Spain one kingdom, at the dawn of the 15th century. From this period we may date the psychical progress of the peninsula.

Although the scriptures were early translated into Spanish, a blind fanaticism overspread the land. The psychical character partook of the heat of her climate, especially the first prose of Alonzo, and the poetry of the monk Gonsalvo, and the composition of the Sequidilla and the Spanish ballads.

The "Guerras de Granada" was the first model of the historical novel, in which De Hita has preceded Scott, in making his hero of an enemy. The lives of Gonsalvo de Cordova, of the Cid, and of other heroes, evince the full conception of knight-errantry, which the racy romance of "Don Quixote" so unmercifully attacked, and with almost as much success as the Don himself did the wine-skins.

We trace the scope of the Spanish genius through a succession of sparkling and imaginative works; those especially of the voluminous dramatists, Lope de Vega and Calderona; of De Leon, the first Spanish ode writer; of "Boscan and Garcilasso," over which Don Juan pored; the racy novels of Lazarillo de Tormes and Guzman de Alfarasche; and the exquisite portraiture of Murillo and Velasquez.

Suddenly, in the 16th century, a blight came over the psychical march of Spain, chiefly the work of Cardinal Ximenes. The Inquisition arose with all its horrors, and freedom of thought and pen were fettered and arraigned. Not only did the flames destroy her sons, but all their valued stores of literature. So the 17th century was a blank in Spanish psychology, and so also will be the 18th.

The psychology of the oriental nations points to India as one of the most interesting studies, as it is a sort of type of the higher intellect of Asia. Its intellectual character, if we may judge from their ancient records, has not progressed from the time of Megasthines—the period of her greatest learning being as far back as her earliest authentic history. The science of metaphysics is profoundly treated in the Sanchyá and Nyayá systems: in the former, natural philosophy and the infinite mind of its author. But it is too transcendental, especially that termed the Yoya philosophy. It is enough, however, combined with the beauties of its poesy, to prove the deeply imaginative minds of the ancient Hindus.

In the Vedanta philosophy we are presented with that beautiful fiction of transmigration, which, we might believe, more than a score of Martin's acts, would inculcate and ensure a freedom from cruelty to animals. They who believe that the souls of their relatives and friends pass into the bodies of the brutes, will be loth to treat them harshly, but would rather cherish and protect them. This is one of the charities that almost consecrates superstition. The psychology of India has been *retrogressive*. Her wealth and her fertility have attracted, from the earliest ages, the inroads of the invader. She has been conquered and enslaved in consequence of her division into many small states, which either spontaneously warred with each other, or were inveigled into opposition by the more powerful victor; and thus, on the principle of "*divide et impera*," Hindostan still remains a sort of psychological problem.

The nature of climate, and other influences, cause the ethnological characters of the Hindus and the Persians to approximate. Their superstitions and their languages are also in close assimilation. The Zend and the Pali dialects are parallels of the Sanskrit and Prakrit of the Brahmin. The solar theism also is common, in varied degrees, to both countries. But we may extend this parallelism to countries remote from these Oriental climes; the worship of Belus at Babylon, and of Osiris in Egypt, suffice to associate the superstitions and theology of nations. The Mosaic authority may be said to corroborate this belief, as it refers to the resemblance of the two great monarchies in the world, Elam and Egypt, the Persians and Hindus being the people of the first. It is by these psychical resemblances that we are drawn imperceptibly nearer to the illustrations of the Mosaic cosmogony. So that the prosecution of ethnology, the Oriental researches of Layard, and the wondrous unfoldings of paleontology, will progressively tend to reconcile those historical discrepancies which have been the stumbling-block of the divine and the triumph of the sceptic.

Egypt, throughout its various transitions, has imparted its character to the surrounding countries—to those of Phœnicia and Chaldæa especially, its theology and its astronomical science. The Arabs were once enlightened, and, we are told, mighty mathematicians. Some, however, even before the birth of Islamism, were, like the Jews, a wandering tribe, and possessing no fixed residence. The Bedouins lapsed into such a state of unintellectuality, that it was termed their "*time of ignorance*," passing their years, like the Scotch borderers, in inroads and maraudings. They became gross idolaters, and lived in sexual socialism, a state which, combined with errant habits, afforded no scope for psychical advancement. The fixed tribes, however, adored the shrine of Mecca, once the dwelling of Abraham and Ishmael, who was probably their primal ancestor, and even prided themselves on their literature, and especially their orations, which are stated to have been highly epigrammatic. At Ochadh they held an annual poetical exhibition, and the Modhahabat, or Moallakat, and the "golden verses," were hung up in the *Caaba*. This *profanation* was abolished by Mahomet, and he gave them in lieu the holy war. Their chief literary glory and inspiration was the wondrous tale of Antar, a hero equal to Hercules or Achilles. It was written by Asmael in the reign of Haroun Alrashchid, about the æra of Charlemagne. It is highly extolled by Sir William Jones, who affirmed it to be very superior to the thousand-and-one nights, as a true picture of Arabian life. Their name now designates a people that overrun the deserts of two quarters, even as the Jew compasses the financial area of four quarters of the globe. In a civilized sense, therefore, an Arab is *but* a name, although the Semitic or Syro-Arabian language, together with the Hebrew tongue, are placed by Schlegel at the summit of the *dialectic* pyramid.

The pyschical history of intellectual nations may be located within a limited circle, if compared with the superficies of the globe, of which the shores of the Mediterranean, and especially the *classic* lands, are the southern bounds, the chief abode of the Iapetidæ of Latham. The complete psychical history of the varieties of man would here require, that one should trace step by step the alphabets and the synonymes of languages, that we might accredit each nation with its due influence in the progress of intellectuality and the advancement of art and science. We do not *now* wish to trench on the field of philology.

There are, however, casual or artificial causes sympathetically stimulating or depressing the functions of the brain; and others, above all, educating the mind, and regulating and curbing the will of man; all exerting a potent influence over the psychical progress of nations.

The habits, customs, manners, and amusements of a people depend somewhat on their temperament and capacity, and are constantly modified by the nature of their locality.

Among the lower races, customs and amusements often approximate closely to the pursuits of their animals. The Ethiopians, and some of the Malays and Mongols, display in both extreme degradation. Their amusements are licentious and brutish, and their occupation a tissue of cruelties. The American is still an *animal*, but of a less ignoble nature—his habits, however, tend to degrade the mind and heart—his war dance and his scalping inspire in his brain the most infuriated ecstasy; revenge and cruelty for ever rankle in his heart. Even the course of wooing is stained by such an impetus; the affection and accomplishments of a lover being measured by the squaw according to the number of victims he has scalped. The habits and amusements of the northern Mongols are marked by slothful luxury. The Esquimo, as he is a swinish feeder, lives in a *sty*, and *maunders* away his useless life. The Chinese Mongol is of course more refined and *intellectual*, but his sloth and sensual luxury must ever be a bar to high mental expansion.

Intercourse with more civilized nations may ameliorate the sensuality of these beings; but the innate propensities will not be subdued, especially if they have been encouraged in early life. We know the impressiveness of a tender mind, and how difficult it is to eradicate the effect of baneful example and tuition.

The Swiss boy has been bred to the mountains; the islander, to the ocean; their habits and their pastimes will of course be those of cragsmen and fishers. The amusements of the town-bred will be the theatre, the concert, the debating-society, and the club-room; and society and fashion will finish every one off as members of one great family.

The pride of birth, rank, and wealth, however, steps in constantly to prevent the law and course of Nature. This exclusiveness imparts a certain psychical character to classes at once to be recognised by an accurate observer.

L'esprit du corps, too, is often ridiculously influential in giving *tone of mind.* Every one appreciates, and often *puffs* over-much, the pursuit in which he is an enthusiast; underrating, often, pre-eminent talent engaged in other callings. The French dancing-master was astonished that Queen Anne had made Harley, Earl of Oxford; for he had thrown away two whole years on the dolt, and could not then teach him to dance.

New lights sometimes burst suddenly on a land, which at once metamorphose the national mind. Thus, at the end of the fifteenth century, on the conquest of Constantinople, the Greek and Latin manuscripts deluged the western world. This, coupled with the ecclesiastical movement of the day, poured out a cornucopia of learning into Italy. Dante, and others under Cardinal Bembo, founded the classic school. New views of geography, thus imported, led to the doubling of the Cape, and the discovery of America.

The Reformation also altered the psychical character of Europe at once, by creating party, and that of the most determined nature: papacy and protestantism were the topics of the day. The religious prejudices of the reigns of Francis, of Charles V., of Henry VIII., of Mary, Elizabeth, and the Stewarts, were the reigning spirit of the time. The one fulmination of the Pope against Luther was the bursting asunder of the psychical bondage, in which the Vatican had held the general mind.

The essay of Milton may be considered almost as the first propounding of that principle which, by the detention of Hampden, and Pym, and Cromwell, in England, in the end established the Commonwealth.

"What great events from trivial causes spring."

The memory and contemplation of genius and virtue,—the graven images of great men, as in the Ceramicum and the Valhalla,—the heroic poems, laudatory of valour,—all these have exerted much influence in elevating and ennobling the national mind. For emulation is not envy: a noble mind aims at superiority, because it *admires* the excellence of a rival.

History and biography at once excite desire of imitation; even songs and common ballads, from the odes of Tyrtæus to the sea songs of Charles Dibdin, have inspired the hearts of a host of heroes. The stateman was shrewd who said, "Let me make the ballads of my country: I care not who makes her laws." The prowess of Themistocles, the graphic force of Thucydides, and the eloquence of Demosthenes, were the result of their excited efforts to emulate Miltiades, Herodotus, and Callistrates.

Although the subject may seem more physical than psychical, yet the sympathies of the stomach with the brain have a powerful influence on mind and character. A *strong* stomach—*dura ilia messorum*—is almost synonymous with success in our pursuits: a healthy digestion cannot be completed without a quiet and unstrained mind. So dyspepsia is a constant penalty for psychical pre-eminence, and pays it off by the re-action on its organ, too often in the end subduing its energy. The effort of study will be often futile immediately after a full meal.

Thus diet, if a *fashion*, may decide the character of a people: the Mosaic code of laws involves the strictest precepts on this point. The rules of medicine, too, were prominent both in Egyptian and Hebrew legislation, and were consecrated as a religious injunction.

Whatever food is defective in its property of assimilation is, in varied degrees, a poison. For if black or unhealthy blood be circulating through the brain, various forms of psychical derangement will be the result. To some new properties imparted to the blood, we may often impute a change of temperament and disposition: the quality of the blood must be congenial with the pursuits of the feeder.

The Spartans, in obedience to one of the precepts of Lycurgus, and the Romans, before the time of Pyrrhus, adopted a very spare but wholesome diet,—they were, indeed, under a constant system of *training*, so that they were ever ready for active exertions. This austerity, perhaps, decided the action of Thermopylæ.

It is recorded of the Bosjesmen, that their character has been changed with their diet: when they led the life of wild shepherds, and fed on roots and larvæ, their habits were passive and apathetic; when they became hunters and flesh-feeders, they also became ferocious and cruel. The Arab, from his own confession, was also cruel and malicious, which physicians assure us was the effect of feeding on camel's flesh.

The Tartar and the Cossack, who drink the blood of wild horses, and the cannibal, who quaffs that of his fellow-men, are marked by predatory and brutal propensities. The Rajpoots, also, are very gross and sensual feeders, and their habits correspond.

We must, however, regard this with reservation. The Samoeids and Ostiaks, who are also blood-drinkers, are dull, sluggish, and slavish. The blubber-eating Esquimo is an almost unintellectual sloth; his mind well-nigh a blank. But these are Mongols of a low degree: while the Chinese, who drenches himself with tea, is also cruel and treacherous; but he inhabits a warmer clime. Here we see organization and climate both influential.

Then the Irish labourer, who feeds on potatoes and water, can endure fourteen hours' labour, and his pugnacious qualities, the effect of whiskey, certainly are not to be questioned.

But the truth is, there is a concentrated energy in the process of assimilation in beings of low intellect. There is no strain of mind to draw off blood from the chylopoietic functions; so the *animal* force bears sway, and a high per-centage of nutriment is abstracted, as in the brute. When Columbus was in Cuba, he found the natives in a state of extreme ignorance. A handful of maize or cassada bread was enough for a meal, so that the Spaniards, the most spare feeders in Europe, seemed to them like cormorants.

The modern Pythagoreans, yclept vegetarians, assure us that their bodily power is increased, and their intellectual faculties rendered lucid, energetic, and undisturbed, under their system. And we are reminded that the diet of the Greeks and Romans, in their palmy days, was chiefly vegetable; but then that character did not change when they adopted animal food. As man is carnivorous, however, and constantly doomed to migrate, he can feed with impunity on the productions of every clime. He therefore wisely adopts a mixed dietetic rule, to suit himself to the torrid zone, in which the extensive consumption of flesh food is precluded by the rapid decomposition of animal fibre; and to the northern latitudes, in which the scantiness of vegetation reduces the inhabitants to diet of more gross and unctuous nature.

We cannot therefore impute an important psychical influence to diet, so long as the system is not physically disordered.

The imbibition of alcoholic liquors, however, comes under a very different category. Ben Jonson's Canary, and Sheridan's Burgundy, as well as Coleridge's opium, for a time inspired *their* intellect, we are told, but it *might have* produced more golden fruit without them. We are not aware of any peculiar people, who, *as a nation*, are topers; yet Tacitus does refer to the custom of very free drinking among the ancient Germans, who quaffed strong drink even to the manes of departed friends. The abuse of opium exerts a very baneful psychical influence. The full or elysian dose is totally subductive of mental integrity; life is a baseless vision that *does* 'leave a wreck behind.'

Those who have witnessed the slaves of the habit, in the divans of Constantinople,—for here the vice is a national one,—will not forget the pictures of psychical derangement—of mental annihilation. From these narcotized Orientals, therefore, we can expect no fruits of intellect. Their field of literature is well-nigh a desert; while they have often been made the slaves of those northern conquerors, whose degree of *natural* intellect was far beneath their own.

The Christian, who knows that his life is one of probationary *denial,* does not thus fall into the pit by wholesale. The whole life of the Moslem, however, is one voluptuous dream—his heaven, the end *for which* he also *lives,* is the paradise of harlots.

But more than all does legislation influence and mark the progress of mind, as it is the bond that unites a community.

The first law was a divine injunction. Religion taught by the tables of Moses was the basis of all subsequent legislation. To love God was the first great law; to love our neighbour, the second.

If these laws were carried out to the letter, pure philanthropy would be the leading spirit of the time: art and science would be secondary; for self-interest, the most potent stimulus to exertion and invention, would be wanting. The age of gold would be established without gold being thought of. *Mind* would be eclipsed by *heart;* and intellect, as regards its deepest study and its loftiest flights, would lie dormant and fallow. But insanity would be a most rare phenomenon; for its essence often consists in over-working of the mind.

This, however, is an Utopian vision. Religion is prone to degenerate into fanaticism and superstition, legislation into despotic and selfish government, and liberty dies when she degenerates into licentiousness and rebellion. Hence the various shades of psychical derangement, even insanity on religious points. Not that religion *creates* insanity (a common error among pseudo-psychologists), but that the excited brain is blinded to its real truths by an *ignis fatuus.*

It is when religion and law are thus set at naught that the most extraordinary psychical phenomena are elicited; the motives of human action being self-gratification and not God's law. Of this truth the deluge and Babel, and Sodom and Nineveh, are historic records.

The revolutions of false religion are less overwhelming and violent than those of the State, but they are not less in perverting the constitution of the mind.

We need but to look on the idolatry and licentiousness of the heathen nations, and on the grovelling propensities of the slave, to be sensible of the benignant influence of Christianity and good government.

The earlier inhabitants of the world were without this light, when the posterity of Seth, proud of their alliance with the women-angels, committed their heinous crimes; the flood came, and a new race, chiefly of Assyrians, Chaldæans, and Hebrews, arose from the sons of Noah. Thus pride of birth destroyed the first people; pride of wealth the second—the full penalty paid for all their psychical debasement.

Among the ancient theists and polytheists, ere the light of revelation beamed abroad, the systems of ethics may indeed have evinced a high refinement of intellect, and the wise men of Athens and of Rome, the learned pundits of Benares, the Peruvian incas, the Mexican caciques, nay, even the skalds of the Hyperborean seas, evolved their various flights of very ingenious mythology. Of their legislation, the codes of Zoroaster, Lycurgus, Solon, and Justinian, are on record. Even among these heathen moralists we certainly learn of high examples of heroic virtue and sacrifice. Vice was distinguished from virtue, and the heathens, even those of the wild races, were impressed with the vision of the Judgment. But truth was yet unrevealed, and the intellectual devotion, without the light of Christian influence, was perilous almost in the direct ratio of its intensity—its children being the spoiler, the sceptic, and the refined voluptuary. Hence, with all this seeming virtue, the mind was absolutely enslaved to vice.

The archives in the temples of heathen Greece, and the pagodas of the Brahmins, were, it is true, enriched by rolls of disquisitions on the distinction between virtue and vice, and their choultries thickly scattered over their land for the reception of the wayfarer. But the virtues of the Stoics were recorded in blood; suicide was deemed the acmé of magnanimity, and a dying gladiator was looked on with a degree of heated enthusiasm, only exceeded by that which her Majesty of Spain displays when a bull rips up the belly of a tauridor. The suicide of the Stoics was a murder—*felo-de-se*—differing from the majority of suicides among the Christians. The effect of the first was to *prevent* insanity—so rare a malady among the heathen; that of the second is, we believe, almost invariably the *result* of a *degree* of madness—the result of those sensations from which the Stoic would release himself by plunging cold steel into his thorax.

As a type of the inconsistencies of a Budhist pagan, we read in the Baghvat Geeta this sublime sentence: "The man is praised who, having subdued all his passions, performeth with his active faculties all the functions of life, unconcerned about the event." With all this fine morality the abominations of their priesthood were almost incredible; and indeed it is probable that we do not yet know all the crimes committed behind the mysterious veils of their gorgeous but polluted temples. The isolation of the Chinese, a nation of sceptics, conceals from us many crimes of which we implicitly believe them guilty. In Thibet, the custom of polyandry, or community of husbands, indicates little modesty in her daughters. Vice must of necessity be predominant in those heathen lands, where the attributes of the Olympic gods and of the idols of the Hindu temples are in the lowest degree licentious and disgusting, and the possession of every vice as it were deified—the base example of the gods consecrating the deeds of their worshippers. We cannot, therefore, believe the account of Marco Polo, that the high Asiatic Mongols and Tatters were chaste to a degree from the influence of their religion, *half* Christianity, or *Shahmanism*. The slavery of this people to the priesthood was equal to that of the low papists at the present time. The Soodar was directly put to death if he but opened the sacred books of the Vedas, as the low Irish suffer a moral death when they are debarred from the life of the Gospel.

The bondage of a false religion is ever wofully detrimental to the progress of intellect, charity being its purest and its noblest manifestation, whether it be of the pagan or jesuitical idolater. The blasphemous sensuality of the Hindu temple, the blind and mad sacrifice of Juggernaut, the Sutti, the murder of the innocents, *i. e.*

the female infanticide of Rajpoot, practised at this very hour, and the murders of the Inquisition, as they are utterly void of charity, the only virtue that can complete the sanctification of a people, must be a fatal error.

To what maniacal enormities has it not given origin, especially in the eleventh, thirteenth and fourteenth centuries. The Vokels, the Fakirs, the Flagellants—Jewish, Catholic, and Protestant persecutions without number, merely because one believed himself right, and therefore all others wrong, forgetting that there may be *two* rights, according to the conscience of a creature. In modern Spain, Portugal, and Italy, the intellect, as a rule, is in a state of apathetic slumber. In France there are a few exalted and leading spirits; the mass presents a painful contrast. Yet Mahomet has been even a greater enemy to psychical improvement than Loyola; the Arabs, once enlightened in art and science, dwindled and decayed under the blight of Islamism. But though the cloistered priest must be ever a bigoted book-worm, yet perhaps Jesuitism effected some counterbalancing good, by inciting our Protestant colleges to greater exertion—even as the present pontiff, by his late appointment, will minister, we hope, to the purification of the Anglican Church, and tend to ensure that Christian benevolence which springs alone from pure and *holy* motives. Else were that standard of excellence vain, which cast down the degraded idols and altars of paganism, and, instead of the mythological creations of a prurient imagination, established divine truth, a truth which has enlightened even the barbarian, if humility has *predisposed* him, for those Laps and Fins who immediately bordered on Russia and Sweden embraced the Christian faith.

But the national mind is too often a proud spirit. The pride of sect is far more detrimental than the pride of birth or wealth, for it condemns nine hundred and ninety-nine out of every thousand of the human race: and yet this is termed religion.

The Christian faith, as it has been desecrated often by national illusion, has often, though failing of its primal object, given character to the spirit of a people. The crusade, for instance, "the first European event," as Guizot terms it, tended to expand the national mind of Western Europe by travel in other lands, and by the accrediting of embassies from Christians to Pagan courts, and by the incitement to discovery. The history of the crusades first roused the curiosity of Marco Polo, and, through his marvellous stories, of Columbus.

As the martial glories of pagan Greece and Rome were the subjects of her wondrous epics, so were the crusades the spring of our later romantic poesy. The adventures of the holy wars have been sung by the poets, from Tasso to Scott: and as English, French, German,

Italian, Spanish, all took part in the martial pilgrimage, a tinge of the romantic was directly given to the literature of those lands, and the poet has consoled himself for the failure of the three great expeditions for the elevation of the Cross.

The second great European event that sprang from a devotional source—the Reformation—metamorphosed for a time the national mind. Wickliffe, Luther, Zwingle, Huss, were the topic of their day, and the pen was engrossed with doctrinal theology, and a purer religion was adopted, although during the onset the flames of the Inquisition certainly rose the higher.

In Germany, France, and England, even to the reign of William the Third (we may to a degree add, to our own day), sectarian theology gave a colour to the psychical character of those countries. In burnings and bloodshed, and the thirty years' war, the minds both of Catholics and Protestants were equally bigoted, and the writings of Spinosa and Socinius tended as much to sap theology as the doctrines of Pyrrho himself.

If we reflect on cosmogony, we find that the religion and theology of a nation were often intimately connected with its government. Religion has not only characterized a nation already established, but it has been the *origin* of a *new* state. In the reign of Elizabeth a handful of puritans wandering from England to Holland, and thence to Massachusetts and Connecticut, founded those states, and established that one religion which now prevails throughout the Transatlantic Union.

The principle of church and state is of high antiquity. The patriarchs were both the kings and priests of their family. Moses and Aaron were the joint rulers of the state. In ancient India, however, the Brahmin was even of the first caste, the monarch of the second; and this Brahmin, like our chancellor, was the conscience-keeper of his sovereign.

The national mind was thus especially prone to take its hue from the example or character of its rulers: just as it follows a fashion set by a great personage; or as the students stained their faces yellow that they might resemble their master.

Moses, Lycurgus, Ptolemy Philadelphus, Pericles, Justinian, Ackber, Alfred, Peter, Napoleon, all imparted their own great conceptions to the national mind, and even to neighbouring nations. So enlightened, indeed, was the reign of Ackber, that even the Greek sophs went to India purposely to converse with the pundits of the holy city of Benares: and as Robertson has hinted, perhaps the stoic school may have had its spring from the notions thus imparted to Zeno and Epictetus. The Peruvians, who had from very high antiquity continued

*in statu quo,* were speedily formed into a government by the genius of Manca Capac, the first Inca.

Hence emanates a passion for learning throughout a state. And this especially, if the one great mind be a patron of art and science. To Mecænas, Pericles, the Medici, are we indebted for many of those splendours which still delight the eye and the understanding of the world. Were such minds in constant succession we may believe to what a degree of perfection the psychical condition of nations would arrive.

But let us take the contrast of these bright pictures—the worst form of government, probably, of which we possess a record, that of Muley Ismael, of Morocco. They must be degraded slaves indeed who, after he had killed forty thousand of his subjects with his own hand—barefooted and trembling, and bowing to the ground, screamed out, "Great is the wisdom of our lord! the voice of our lord is like the voice of an angel from heaven."

With the contrasts of the psychical effects of servility and freedom on a country, history abounds, not only regarding the happiness and civilization, but the intellect of a people. In the one state we may find, perchance, a few leading minds, meteors that blaze for awhile amid a host of slaves; in the other, science is spread abroad, and as a rule, remunerated *ad valorem.*

In Russia, for instance, the bondage of the serfs causes the manifestations of intellect to be very low and rare indeed.

In the servility of rebellion (the rebel despot being the most cruel of all the slaves of power) the intellect of a people must of necessity become degraded, and degenerate into a national monomania. They may cringe and bow awhile to their idol, until the crushing of his iron law causes the worm to turn and sting the tyrant to death. Then comes the reign of anarchy. The republican mind will be too much absorbed in political disquisition, or intestine warfare, to even dream of those pursuits which enlighten the mind and soften and amend the heart.

But even *legitimate* warfare, as it is termed, is too often the vain-glory of a nation, to the blighting of its psychical progress; although it is true many illustrious men are deeply associated with war, but they have deemed it a necessity, not a glory. Fabricius, Cincinnatus, Washington, grasped not an imperial crown, like the first Consul of France, and therefore they are glorified while Napoleon was reviled and hated. The history of a nation, therefore, is too often a history of warfare, the high Mongol dynasty, perhaps, excepted. With the American, the Æthiopian, and the Malay, war is a *passion,* the indulgence of malevolent revenge. There is no quarter; for they fight to kill, not to conquer; the prisoner of war is not immured and exchanged, but scalped. If wounded, like the Feræ, they tear the weapon from their flesh, as Herera and others report, and break the shaft and dash it with execrations on the ground. Their victories are the result chiefly of stratagem, or a sort of instinctive cunning, aided by the acuteness of their *physical* sense. They have, it is true, a sort of discretion, for they *run away* if they are likely to be worsted. In this too they resemble the Feræ, as they do indeed even in their feeding, after which they are apathetic, but when hungry or aroused they then play the tiger. With the Caucasian (the Iapetidæ) war is a *science*, a system. We see the *plan* of a battle, the *co-operation* of forces—a word, which in itself implies civilization, and advance of psychical capacity. The first grand step of the Romans was in arms, especially after the fall of the Tarquins, when the annual elections kept the leaders on the *qui vive;* and in the wars with Pyrrhus, especially as the consulship and ovations were the guerdon of military prowess.

From the earliest æra the mind of man has run wild upon invasion, and he has jumbled together religion and wholesale murders, making the former the pretext for the latter; the common psychical development was desire of conquest. The holy wars of the Crusades—the conquests of Mahomet, the three changes of dynasty in Constantinople, were some of the monomaniacal illusions which from time to time have controlled and metamorphosed the psychology of the world.

And such was its psychical influence, that the *peace of war* became the pastime of the nobility—especially after Philip the First of France, in the lofty spirit of chivalry, established the *joûte* and the *tournoi.*

But contention on a field is the grave of intellectuality. Even the sçavans of Napoleon, with all their boasted researches in Egypt, came meagre off—if we except Denon, and he would have done better in peace; and Larrey, who threw some light on military surgery—but even that is a poor compensation for the loss of legs and arms and lives.

What lesson may we learn, then, from our brief psychical survey of the globe? That intellect is vain if we promote not the moral happiness of man. He should have but one idol—his Maker; but one motive—a Christian spirit of benevolence.

We cannot hope, of course, for a community of taste in art and science, but we *may* hope that the foreshadowed national intercommunication will ensure a bond of sympathetic interest between the four quarters of the globe, if it do not in the end establish an Utopia of the universal mind.

